# Towards the theoretical limitations of X-ray nanocrystallography at high intensity: the validity of the effective-form-factor description

**DOI:** 10.1107/S2052252518011442

**Published:** 2018-09-13

**Authors:** Malik Muhammad Abdullah, Sang-Kil Son, Zoltan Jurek, Robin Santra

**Affiliations:** a Center for Free-Electron Laser Science, Deutsches Elektronen-Synchrotron DESY, Notkestrasse 85, 22607 Hamburg, Germany; b The Hamburg Centre for Ultrafast Imaging, Luruper Chaussee 149, 22761 Hamburg, Germany; c Department of Physics, University of Hamburg, Jungiusstrasse 9, 20355 Hamburg, Germany

**Keywords:** XFELs, X-ray nanocrystallography, effective form factor, ionization, radiation damage

## Abstract

X-ray free-electron lasers (XFELs) broaden horizons in X-ray crystallography. Facilitated by the unprecedented high intensity and ultrashort duration of the XFEL pulses, they enable us to investigate the structure and dynamics of macromolecules with nano-sized crystals. A limitation is the extent of radiation damage in the nanocrystal target. A large degree of ionization initiated by the incident high-intensity XFEL pulse alters the scattering properties of the atoms leading to perturbed measured patterns. In this article, the effective-form-factor approximation applied to capture this phenomenon is discussed. Additionally, the importance of temporal configurational fluctuations at high intensities, shaping these quantities besides the average electron loss, is shown. An analysis regarding the applicability of the approach to targets consisting of several atomic species is made, both theoretically and *via* realistic radiation-damage simulations. It is concluded that, up to intensities relevant for XFEL-based nanocrystallography, the effective-form-factor description is sufficiently accurate. This work justifies treating measured scattering patterns using conventional structure-reconstruction algorithms.

## Abstract 2

This article presents a discussion of the generalization of the effective-form-factor approximation applied to describe scattering patterns with a severe degree of ionization and a demonstration of its applicability to X-ray free-electron laser (XFEL)-based nanocrystallography *via* realistic radiation-damage simulation of nanocrystals.

## 1. Introduction   

X-ray free-electron lasers (XFELs) (Berrah & Bucksbaum, 2014 ▸; Emma *et al.*, 2010 ▸) provide ultrashort X-ray pulses with unparalleled luminosity, producing ultrabright diffraction patterns, which enable atomic scale reconstruction of bio­molecular structures. Unraveling the structural changes in XFEL-irradiated biomolecules has evoked great interest for decades (Neutze *et al.*, 2000 ▸; Chapman *et al.*, 2011 ▸; Boutet *et al.*, 2012 ▸; Redecke *et al.*, 2013 ▸). Recent advances in the technology of X-ray sources have opened new horizons in the field of time-resolved X-ray crystallography. According to a novel experimental scheme called serial femtosecond crystallography (SFX) (Chapman, 2015 ▸), biomolecular nanocrystals are individually illuminated by one XFEL pulse each and the scattering patterns are recorded. A set of such patterns is then used for the determination of the electron density. However, an XFEL pulse also induces radiation damage in the targeted sample.

Numerous theoretical and numerical studies have been carried out in order to investigate the effects of radiation damage in single instances and nanocrystals of biological molecules (*e.g.* proteins) and also in non-biological systems (Murphy *et al.*, 2014 ▸; Abdullah *et al.*, 2016 ▸, 2017 ▸; Neutze, 2014 ▸; Curwood *et al.*, 2013 ▸; Chapman *et al.*, 2014 ▸; Ziaja *et al.*, 2015 ▸; Ho *et al.*, 2016 ▸). X-ray irradiation causes electronic damage, which affects the atomic form factors (Quiney & Nugent, 2011 ▸; Son, Young & Santra, 2011 ▸) and may also result in atomic displacements on longer time-scales, leading to the annihilation of the Bragg diffraction spots (Barty *et al.*, 2012 ▸).

Ionization reduces the atomic form factors and therefore the scattered signal and thus limits the achievable resolution. In order to distinguish the atomic species of a nanocrystal from reconstruction, the atom type may need to be assigned on the basis of the electron density. Atomic species like carbon, nitrogen and oxygen, which exist in large numbers in biomolecular crystals and have similar atomic numbers, may be difficult to distinguish because of electronic damage. Furthermore, as the patterns accumulate signal from a sample undergoing radiation induced changes, they are no longer in a strict Fourier-transformation relationship with the electron density. Therefore, theoretical investigations are essential in understanding the formation and information content of such non-ideal patterns.

In one approach, it is considered that scattering possesses the statistical characteristics of a partially coherent diffraction pattern (Quiney & Nugent, 2011 ▸; Lorenz *et al.*, 2012 ▸; Hau-Riege *et al.*, 2007 ▸), whereas in the case of molecules containing a single atomic species and assuming a simple linear scaling relation between charge state and atomic form factors, the scattering pattern can be written as a coherent sum based on effective electron densities (Hau-Riege *et al.*, 2007 ▸). Recently, in the case of large biomolecular species, a simple approximation of using effective form factors defined by the square root of the time-averaged square of time-dependent scattering factors has also been employed (Lunin *et al.*, 2013 ▸; Lunin *et al.*, 2015 ▸).

In this article, we will redefine the effective form-factors, emphasizing the implications for the interpretation of the scattering patterns. A time-integrated pattern does not correspond to a static electron density *via* a Fourier transform in a mathematically rigorous manner. It is formed by an incoherent sum of non-identical, individually coherent patterns. Therefore, it is not straightforward that conventional pattern-processing schemes can be expected to work. However, if the temporal-variance-aided effective-form-factor description is proven to be accurate under relevant damage conditions, it also ensures that the time-integrated pattern can be treated as a coherent pattern to good accuracy, and image processing algorithms can be expected to converge and deliver a solution. By using a realistic radiation-damage model including both atomic and environmental effects, we theoretically investigate the limitations of the simple effective-form-factor concept on the example of a glycine (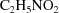) organic nanocrystal. By calculating Bragg intensities we analyze the contribution of the temporal variance and the threshold pulse intensity up to which the constructed effective form factors are valid to describe the non-ideal patterns, thus allowing for the use of conventional crystallography processing methods.

## 2. Theoretical methods   

The scattering pattern of a crystal affected by severe radiation damage at high X-ray intensity is calculated by an incoherent summation over all possible electronic and nuclear configurations weighted by the corresponding probabilities of occurrence at a given time and then accumulated over the whole X-ray pulse. When a crystal is exposed to a high-intensity X-ray beam with fluence 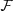 and photon energy ω (we employ atomic units), the scattering intensity at the momentum transfer 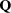 is given by 
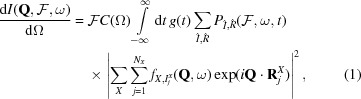
where *X* indicates the atomic species and *j* represents the atomic index of that species. 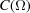 represents a factor depending on the polarization of the X-ray pulse, while 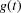 is the normalized temporal envelope of the pulse. We assume a uniform fluence distribution within the irradiated part of the crystal (Abdullah *et al.*, 2016 ▸). 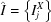 is the global electronic configuration of the crystal, which is given by specifying the electronic configuration 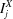 of all individual atoms, and 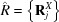 is the global nuclear configuration of all atomic positions 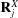 in the nanocrystal. The atomic form factor differs for different atomic species *X* and different electronic configurations, so it is given by 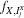. 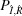 is the time-dependent probability of 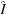 and 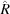, which also depends on 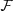 and ω. Note that it is critical to obtain the time evolution of 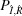 in order to evaluate the scattering intensity of equation (1).

In contrast, the scattering intensity for an undamaged sample is calculated simply by using a coherent sum (the dependence on 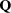, 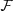 and ω is omitted for the sake of convenience), 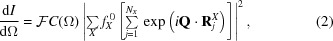
where 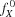 is the atomic form factor of the atomic species *X* in the neutral ground state. Here we consider nonresonant X-ray scattering only.

Our goal is to approximate the scattering intensity for XFEL-irradiated crystals by using a simple coherent form as in equation (2). The simplest solution can be obtained by replacing 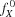 with the time-averaged atomic form factor, 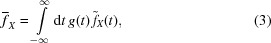
where 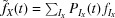 is the time-dependent atomic form factor during the X-ray pulse and 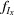 is the atomic form factor of the 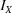
th electronic configuration of the given atomic species *X*. 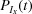 is the configurational population at a given time *t*, which was considered within the independent-atom model (Son, Chapman & Santra, 2011 ▸, 2013 ▸). The time-averaged atomic form factor 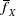 at 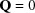 is typically interpreted as the effective charge during the X-ray pulse for the given atomic species. The effective charge (time-averaged electron loss) is enhanced as the intensity increases (see Fig. 2 ▸) because of ionization dynamics, thus reducing the time-averaged form factor.

On the other hand, it has been suggested that the time-averaged atomic form factor is not enough to describe the scattering intensity in the case of high-intensity X-ray fields (Son, Chapman & Santra, 2011 ▸, 2013 ▸; Galli, Son, Barends *et al.*, 2015 ▸). Since the time-dependent atomic form factor varies dramatically during an intense X-ray pulse, the temporal variance needs to be taken into account (Son, Chapman & Santra, 2013 ▸). For a single atomic species, it is trivial to derive the following effective form factor from the generalized Karle-Hendrickson equation (Son, Chapman & Santra, 2011 ▸, 2013 ▸): 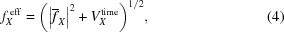
where 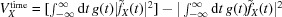. If the XFEL-irradiated crystal consists of more than one atomic species, it can be shown that the scattering intensity may be approximated by a coherent sum as in equation (2), with the effective atomic form factors defined in equation (4) (see Appendix *A* [App appa] for details). With this definition, the distinction between the effective form factor and that derived from the effective charge can be clearly seen. Since 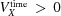, the time-averaged form factor 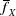 always underestimates the effective form factor 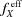. We will present a detailed numerical analysis for those form factors in the following section, based on realistic radiation-damage simulations of nanocrystals irradiated by intense X-ray pulses. Note that the form of equation(4) is equivalent to that proposed in Lunin *et al.* (2015 ▸): 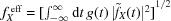.

## 3. Numerical analysis   

### 3.1. Simulation methods   

In order to perform a simulation of a nanocrystal exposed to an intense X-ray pulse, we subdivide the nanocrystal into supercells and simulate the ionization and nuclear dynamics for the supercells using *XMDYN* (Jurek *et al.*, 2016 ▸; Murphy *et al.*, 2014 ▸; Tachibana *et al.*, 2015 ▸), applying periodic boundary conditions. *XMDYN* is a radiation-damage-simulation tool that takes into account the inner-shell processes, such as photoionization, Auger and fluorescent relaxation, as well as phenomena caused by the environment, such as collisional ionization, recombination and dynamics driven by Coulomb interaction between charged particles. This supercell approach of *XMDYN* has been applied before to bulk systems (Abdullah *et al.*, 2016 ▸, 2017 ▸). To construct a scattering pattern from the nanocrystal, we employ the code *XSINC* (Abdullah *et al.*, 2016 ▸).

In our investigation, for each Bragg reflection, *XSINC* analyzes the scattering intensity in equation (1) with 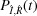 obtained from realistic simulations of *XMDYN*, including both impact ionization (Bekx *et al.*, 2018 ▸) and recombination, which are critical in a dense matter environment (Abdullah *et al.*, 2016 ▸). With 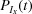 derived from 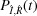, the time-averaged atomic form factor 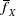 in equation (3) and the effective atomic form factor 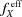 in equation (4) are calculated using *XSINC*.

### 3.2. Results   

**Figure 1:**
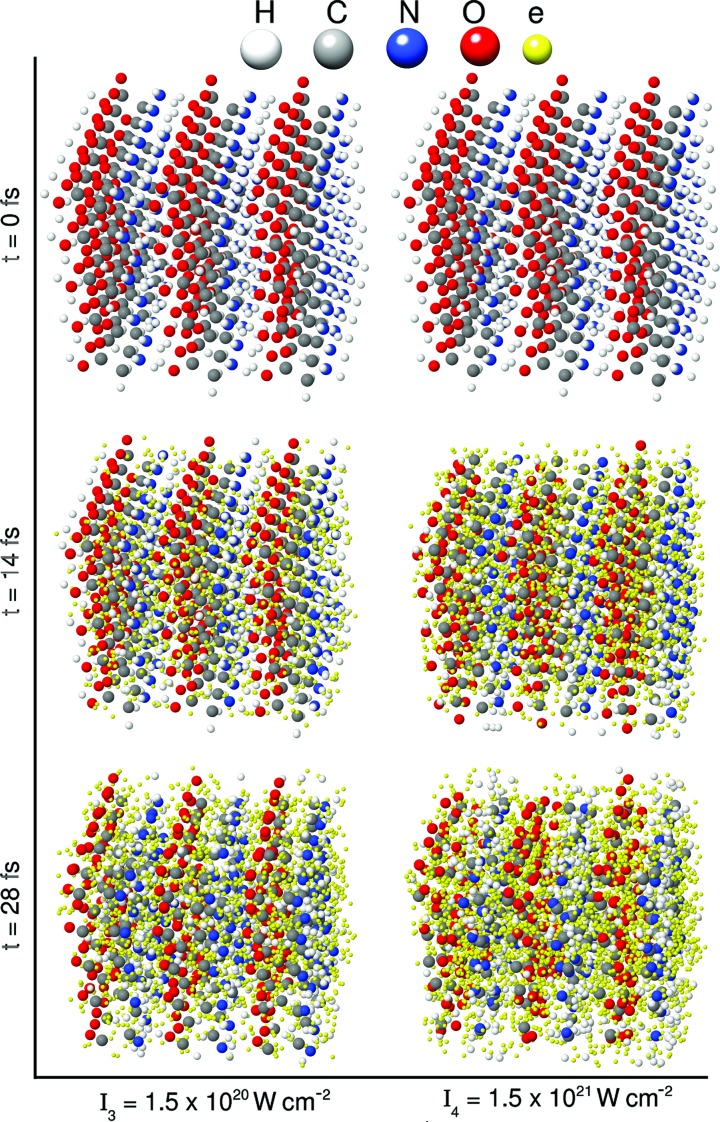
Real-space snapshots of ionization dynamics of a supercell comprising 105 molecules of glycine. The photon energy is 10 keV; the peak intensities are 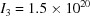 and 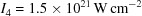. The temporal pulse envelope is Gaussian with 10 fs FWHM. The pulse is centered at *t* = 14 fs.

In our analysis, we considered a nanocrystal of the amino acid glycine. The virtually assembled crystallographic unit cell is orthorhombic with cell parameters of *U*_*x*_ = 5.7248 *U*_*y*_ = 2.986 and *U*_*z*_ = 1.912 Å, containing one molecule. In our simulation, we constructed a supercell with dimensions of *S*_*x*_ = 17.174, *S*_*y*_= 14.93 and *S*_*z*_ = 13.384 Å, containing 105 glycine molecules. For scattering pattern calculations, we considered a nanocrystal consisting of 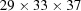 supercells. We used a photon energy of 10 keV and four different X-ray peak intensities: *I*_1_ = 1.5 × 10^18^, *I*_2_ = 1.5 × 10^19^, *I*_3_ = 1.5 × 10^20^ and *I*_4_ = 1.5 × 10^21^ W cm^−2^. The temporal pulse envelope is Gaussian with 10 fs full width at half-maximum (FWHM) and we assumed spatially uniform irradiation. For each peak intensity, 150 *XMDYN* trajectories were calculated. Fig. 1 ▸ shows real-space snapshots of the atoms in a single supercell undergoing ionization as a function of time, for the intensities 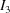 and 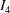. It can be seen from the increasing number of ejected electrons that the structure is substantially ionized by the end of the pulse for the 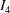 case. However, during the short time duration of the pulse, the atomic displacements did not exceed 0.15 Å for C, N, and O, being well below the resolution. Hence the Bragg reflections are not affected by the atomic movement. The free-electron contribution to the coherent signal is also negligible because of the fairly uniform average spatial distribution.

**Figure 2:**
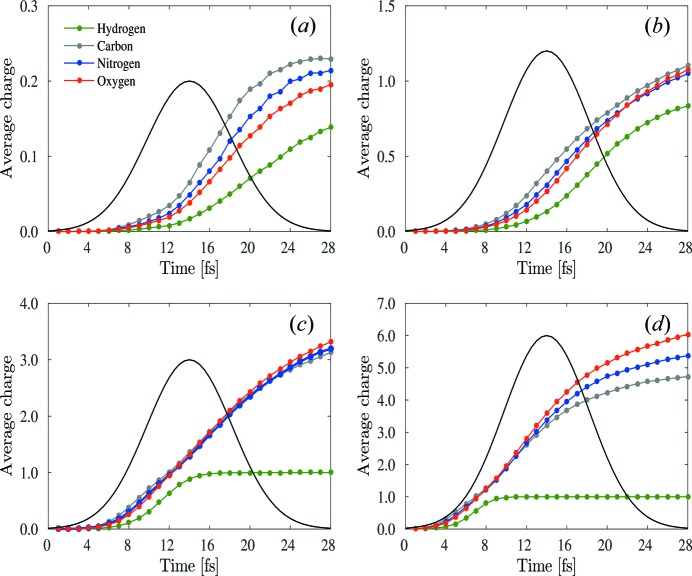
Average charge as a function of time at the intensity of (*a*) 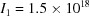, (*b*) 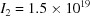, (*c*) 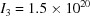 and (*d*) 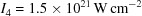. The black curve represents the temporal Gaussian envelope of 10 fs FWHM, centered at *t* = 14 fs.

Fig. 2 ▸ shows the time evolution of the charge for different atomic species at different intensities. For the lowest intensity (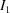), almost all the species remain neutral (charges 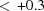) after irradiation, whereas for the highest intensity (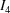), carbon, nitrogen and oxygen are ionized up to charge states of +4.7, +5.2 and +6.0, respectively. To saturate single-photon absorption for light atoms (carbon, nitrogen and oxygen) at 10 keV, the intensity at 10 fs FWHM must be larger than 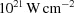. Therefore, X-ray multiphoton ionization does not play a significant role in the intensity regime under consideration, except for the highest intensity. The drastic changes in the charge states shown in the high-intensity cases in Fig.2 ▸ are mainly caused by electron-impact ionization (Abdullah *et al.*, 2016 ▸), resulting in severe radiation damage.

**Figure 3:**
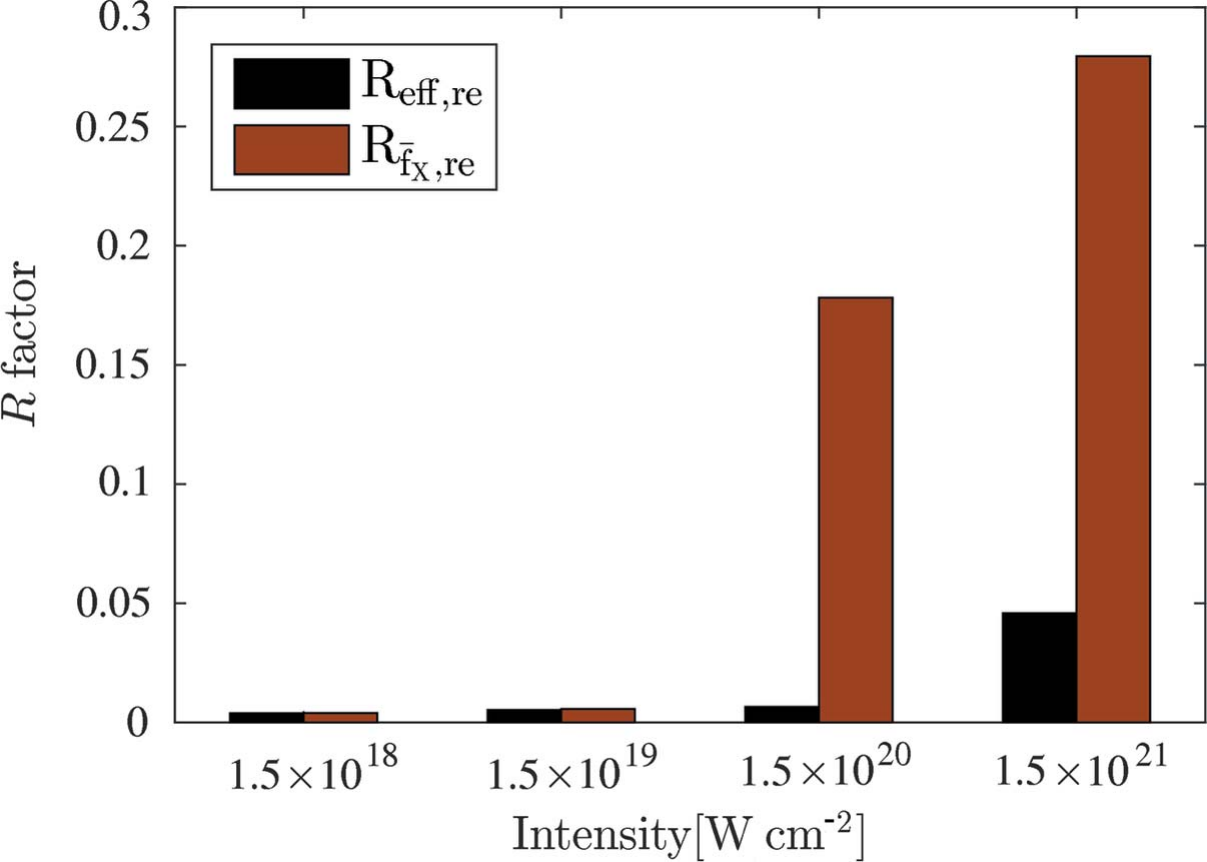
Crystallographic *R* factor in two different cases as a function of intensity. The black bars represent 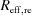, the brown bars represent 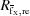.

The accuracy of the effective-form-factor approximation is verified by the crystallographic *R* factor, which is widely used as a measure of the agreement between calculated patterns based on a crystallographic model and the experimental patterns. In Fig. 3 ▸ we compare the goodness of fit of two different approximations using the *R* factor at several intensities. 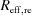 is defined by 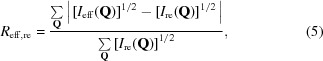
where the real intensities 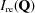 are calculated from the incoherent sum, with full dynamics calculations, in equation (1) and 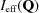 is calculated from the coherent sum in equation (2) by replacing 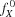 with the effective form factors 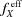 [equation(4)]. Similarly, 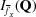 is obtained by replacing 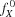 with 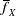. Then, 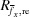 is calculated from 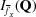 and 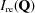. The crystallographic *R* factor is calculated using reflections up to miller index (6 6 6) which corresponds to the resolution of 1.58 Å. The *R*-factor value required for successful structural determination at atomic resolution is suggested to be 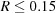 as a rule of thumb (Neutze *et al.*, 2000 ▸), supported also by statistical analysis of deposited solutions in the Protein Data Bank (PDB) (Urzhumtsev *et al.*, 2009 ▸). The minimum possible value of *R* factor is zero, indicating perfect agreement between the considered cases. It can be seen that for the highest intensity (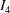), 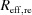 is still only about 0.05, which indicates good agreement between 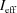 and 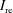. Hence, the coherent sum with the effective atomic form factors used here can describe the radiation damage in a nanocrystal even for the highest intensity (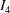). On the other hand, 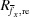 increases much more rapidly as a function of the intensity, indicating that the time-averaged atomic form factor 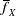 is a poor choice when attempting to approximate the non-ideal pattern in terms of a coherent pattern; 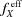 [equation (4)] provides a much better fit, particularly at the highest intensities considered here.

**Figure 4:**
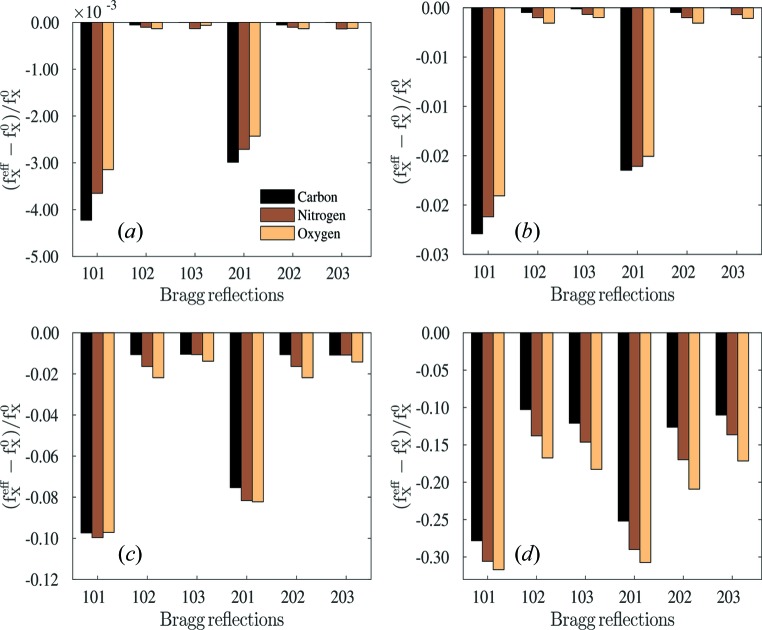
Relative differences of the effective form factor (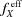) compared with the ideal form factor (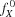) for different atomic species. The peak intensity for each panel is the same as used in Fig. 2 ▸.

To further explore the changes caused by radiation-damage dynamics using the effective form factors, we analyzed the relative difference between the effective and ideal (un­damaged) form factors, 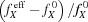, as shown in Fig. 4 ▸. The effective atomic form factors are always reduced because of the radiation damage, so all plots in Fig. 4 ▸ are negative. The relative differences are almost negligible at low intensities (see Figs. 4*a* ▸ and 4*b*), but no longer at high intensities; the maximum difference is about 10% in Fig. 4(*c*) ▸ and 30% in Fig.4(*d*) ▸. Moreover, these relative differences are not constant for different Bragg reflections and different atomic species. For example, at the lowest intensity in Fig.4(*a*) ▸, the effective form factors of carbon at the (101) and (201) reflections are more reduced than those of oxygen, even though the percentage is very small. At the highest intensity in Fig. 4(*d*) ▸, the 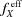 of oxygen are more reduced than those of carbon, and the relative differences fluctuate between 10% and 30% for different Bragg reflections. Hence, the effective form factors cannot, in general, be obtained by multiplying the standard form factors 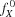 by a single uniform scaling factor.

## 4. Conclusions   

In summary, we have discussed the generalization of the effective-form-factor approximation applied to describe scattering patterns from XFEL-irradiated samples consisting of multiple atomic species. We have shown that these quantities are mainly shaped by the average electron loss caused by stochastic ionization events and dynamical configurational fluctuations. We have demonstrated *via* realistic numerical simulations that the role of the latter contribution becomes more prominent with increasing X-ray intensity. Still, up to intensities relevant for XFELs, the effective-form-factor description is acceptable, also implying that conventional structure-reconstruction algorithms dealing with purely coherent scattering signals can be expected to work in this intensity regime as well.

## Appendix A Effective atomic form factor

First, let us define the effective form factor in equation (4). In a similar fashion to Galli, Son, Barends *et al.* (2015 ▸), the effective atomic form factor is defined by the square root of the scattering intensity given by only one atomic species *X* after averaging over time and configurations:

We assume that the nanocrystal is exposed to a homogeneous fluence distribution (Abdullah *et al.*, 2016 ▸). Assuming that no nuclear motions are involved during the short pulse duration and radiation-damage dynamics of individual atoms happen individually, the global population is given by the product of the individual atomic populations with the corresponding electronic configuration,

We also assume that the dynamical profiles of individual atomic populations are similar to each other for a given atomic species, . Then the effective atomic form factor goes over into

where  (the dependence of ,  and ω is omitted). A similar analysis was performed in Son, Chapman & Santra (2011 ▸). The term within the brackets in equation (8) diminishes when  becomes large, because at Bragg peaks . It is worthwhile noting that this definition of the effective atomic form factor is directly connected to the MAD coefficient  in Son, Chapman & Santra (2011 ▸): .

Next, we demonstrate how the scattering intensity may be approximated by a coherent sum using the effective form factors in equation (2). We start from equation (2) in Appendix *A* in Galli, Son, White *et al.* (2015 ▸). For simplicity, we consider only two atomic species, *A* and *B* (an extension to many atomic species is straightforward):

The above scattering-intensity expression can be written as the extended Karle–Hendrickson equation, following the expressions in Galli, Son, White *et al.* (2015 ▸),

When anomalous scattering contributions are small enough (for example, light atoms at hard X-rays as demonstrated in the main text), the term with  may be neglected, while the term with  still needs to be evaluated because of the coupling of  and . Here the molecular form factor is defined by

and the phase difference is . Note that the dependence on  and ω is omitted for simplicity. The atom-specific MAD coefficients are given by

and the biatom-specific MAD coefficient  is given by

After plugging the effective form factor  into equation (10), the scattering intensity is recast as

where  (Son, Chapman & Santra, 2013 ▸). Under Bragg conditions, the terms with  are smaller than others as  becomes larger. In addition, let us assume that time profiles of the dynamical behavior of different atomic species are proportional to , such that  and . The factor   then becomes

Consequently, if we assume small anomalous scattering signals and similar dynamical behavior for different atomic species, and neglect small  terms, then the scattering intensity may be expressed as the conventional coherent sum,
